# Lymph node ratio emerges as a pivotal prognostic determinant for cancer-specific survival amidst individuals diagnosed with stage N1 and N2 non-small cell lung carcinoma: A population-based retrospective cohort study

**DOI:** 10.1097/MD.0000000000042202

**Published:** 2025-04-18

**Authors:** Xiaowei He, Ying Huang, Tao Hu

**Affiliations:** a Department of Pulmonary and Critical Care Medicine, Wuning County People’s Hospital, Jiujiang City, Jiangxi Province, China; b Department of Pulmonary and Critical Care Medicine, Jiangxi Provincial People’s Hospital, The First Affiliated Hospital of Nanchang Medical College, Nanchang City, Jiangxi Province, China; c Department of Cardiovascular Medicine, Jiangxi Provincial People’s Hospital, The First Affiliated Hospital of Nanchang Medical College, Nanchang City, Jiangxi Province, China.

**Keywords:** cancer-specific survival, lymph node ratio, non-small cell lung cancer, prognostic biomarker, staging optimization

## Abstract

Lung cancer is the leading cause of cancer-related deaths in the US, predominantly non-small cell lung cancer (NSCLC). Lymph node metastasis significantly impacts prognosis, yet current classification systems lack precision. Lymph node ratio (LNR), correlating metastatic to total lymph nodes, emerges as a superior prognostic tool. The study aimed to identify statistically validated LNR cutoffs and evaluate their prognostic significance in NSCLC patients, addressing limitations in the current tumor, node, and metastasis staging system. The study utilized data from the surveillance, epidemiology, and end results database (2010–2019) to analyze NSCLC patients undergoing tumor excision. Exclusions included those receiving chemotherapy or radiotherapy. Patients receiving chemotherapy or radiotherapy were excluded to isolate the independent impact of surgical lymph node retrieval on cancer-specific survival. The primary outcome focused on cancer-specific survival (CSS) stratified by LNR, with secondary analysis on N1/N2 NSCLC cases. X-tile software determined LNR cutoffs, categorizing patients into 3 groups. Descriptive statistics and Kaplan–Meier analysis were employed, along with multivariate Cox regression. Lower Akaike information criterion (AIC) values favored LNR models. Empower Stats and R software were utilized, with *P* < .05 indicating significance. Median CSS follow-up was 22 months, with 1265 NSCLC-related deaths. Lymph node retrieval averaged 11, with a median LNR of 0.15. X-tile analysis revealed LNR thresholds of 0.17 and 0.34, stratifying patients into low, medium, and high-risk groups. Kaplan–Meier show better differentiation when LNR is used as a predictor compared to N staging (LNR *P* < .0001 vs N stage *P* = .91). Multivariate Cox regression confirmed high LNR as an independent predictor of poorer CSS. A lower Akaike information criterion for LNR models highlighted its superior prognostic accuracy over N staging. This study demonstrates that the LNR is a superior prognostic indicator for N1 and N2 stage NSCLC compared to traditional N staging. By integrating metastatic burden and lymph node retrieval extent, LNR addresses key gaps in the tumor, node, and metastasis system and provides enhanced risk stratification. Clinicians can use LNR to identify high-risk patients who may benefit from intensified adjuvant therapies or tailored follow-up protocols. Further prospective studies are warranted to establish standardized LNR thresholds and validate its integration into clinical practice.

## 1. Introduction

Lung cancer remains the leading cause of cancer-related mortality within the United States, with an annual incidence of approximately 200,000 cases and 160,000 deaths.^[1]^ Non-small cell lung cancer (NSCLC) constitutes 85% of the total lung cancer diagnoses.^[1,2]^ Lymph node (LN) metastasis and its distribution in NSCLC patients are critical for prognosis and treatment strategies.^[3]^ Patients with lymph node involvement in the hilar or mediastinal regions often face a poorer prognosis. The current American Joint Committee on Cancer 8th edition tumor, node, and metastasis (TNM) staging system classifies lymph node status based on anatomical location and the number of involved nodal stations.^[4–9]^

The TNM system provides essential insights for tumor evaluation, treatment planning, and prognosis. Lung cancer characterization has evolved beyond anatomical factors to include clinical, biological, molecular, and genetic considerations. Thus, the integration of novel and pertinent factors is imperative to devise prognostic frameworks capable of enhancing the evaluation and therapeutic decisions for patients afflicted with lung cancer.

Systematic lymph node sampling requires meticulous identification, and the identification of positive lymph nodes depends on adequate lymph node retrieval and examination. Moreover, factors such as age, tumor localization, T stage, extent of lymph node dissection, and the scrupulousness of pathological assessment exert notable influence on positive lymph nodes determination. Accurate staging is crucial for providing prognostic insights and guiding optimal treatment for lung cancer patients, prompting a surge in research endeavors aimed at refining staging methodologies for NSCLC.

Emerging evidence highlights the lymph node ratio (LNR)—the ratio of metastatic lymph nodes to total examined nodes—as a superior prognostic indicator in malignancies such as breast, bladder, gastric, colon, and rectal cancers.^[10–14]^ In NSCLC, studies suggest that the number of metastatic lymph nodes provides greater prognostic value than their anatomical location.^[15–17]^ However, the current TNM system’s reliance on anatomical localization of LN involvement (N1/N2 classification) is increasingly recognized as insufficient. This approach oversimplifies the complexity of LN metastasis and fails to account for critical factors, such as the burden of metastatic disease and the extent of lymph node dissection, which can significantly impact prognosis. Unlike TNM’s focus on anatomical localization, LNR captures 2 critical prognostic factors: (1) the relative metastatic burden and (2) the adequacy of lymph node dissection. By integrating these variables, LNR provides a more precise and individualized prognostic framework.

Adjuvant chemotherapy stands as a customary recommendation for individuals afflicted with NSCLC displaying indications of lymph node metastasis, yielding discernible and clinically significant enhancements in survival outcomes. Encouragingly, evidence has emerged bolstering the utility of the LNR as a prognostic determinant specifically within the realm of N1 NSCLC within clinical settings. Previous investigations have elucidated that the incorporation of LNR furnishes clinicians with autonomous insights, facilitating more precise staging and enabling tailored adjuvant therapeutic strategies for NSCLC patients.^[18]^ Patients exhibiting elevated LNR levels confront escalated risks of disease recurrence and merit contemplation for more aggressive postoperative interventions aimed at ameliorating their long-term prognostic outlook. Furthermore, investigations suggest that LNR may serve as a discerning tool in identifying candidates likely to derive survival benefits from postoperative radiotherapy, thereby advocating for its incorporation into clinical decision-making frameworks. These findings underscore the potential of LNR as a pivotal prognostic indicator in NSCLC, portending its forthcoming integration into routine clinical practice. Indeed, LNR emerges as an autonomous prognostic factor offering enhanced precision in staging and facilitating tailored postoperative adjuvant interventions for NSCLC patients. However, it is imperative to acknowledge the limitations inherent in these studies, chiefly stemming from the modest scale of the study cohorts and the methodological intricacies associated with determining the optimal LNR threshold values.

The current clinical guidelines classify lymph node status primarily based on anatomical location and the number of involved stations, but they overlook the potential prognostic value of the LNR.^[18,19]^ Although previous studies have highlighted the independent prognostic significance of LNR in several cancers, including breast, gastric, and rectal cancers, LNR has not yet been incorporated into the staging system for NSCLC. This represents a significant gap. Our study aims to address this gap by utilizing the surveillance, epidemiology, and end results (SEER) database to evaluate the independent role of LNR in predicting prognosis for patients with N1 and N2 stage NSCLC. This study further seeks to refine the NSCLC staging system and facilitate the implementation of tailored postoperative interventions. The objectives of our inquiry were 3-fold: firstly, to determine the optimal threshold for the LNR using the SEER database and X-tile software; secondly, to evaluate the independent prognostic significance of LNR in cancer-specific survival (CSS) among N1 and N2 stage NSCLC patients; and thirdly, to assess the potential clinical benefits of integrating LNR into routine practice. Specifically, we aim to demonstrate that LNR can improve risk stratification of NSCLC patients, allowing for more accurate identification of high-risk individuals who may benefit from intensified postoperative management, including tailored adjuvant therapies such as chemotherapy or radiotherapy. Ultimately, our study seeks to refine the TNM staging system to provide clinicians with a practical tool for individualized patient care and better long-term outcomes.

## 2. Methods

### 2.1. Data source

**Figure 1.**
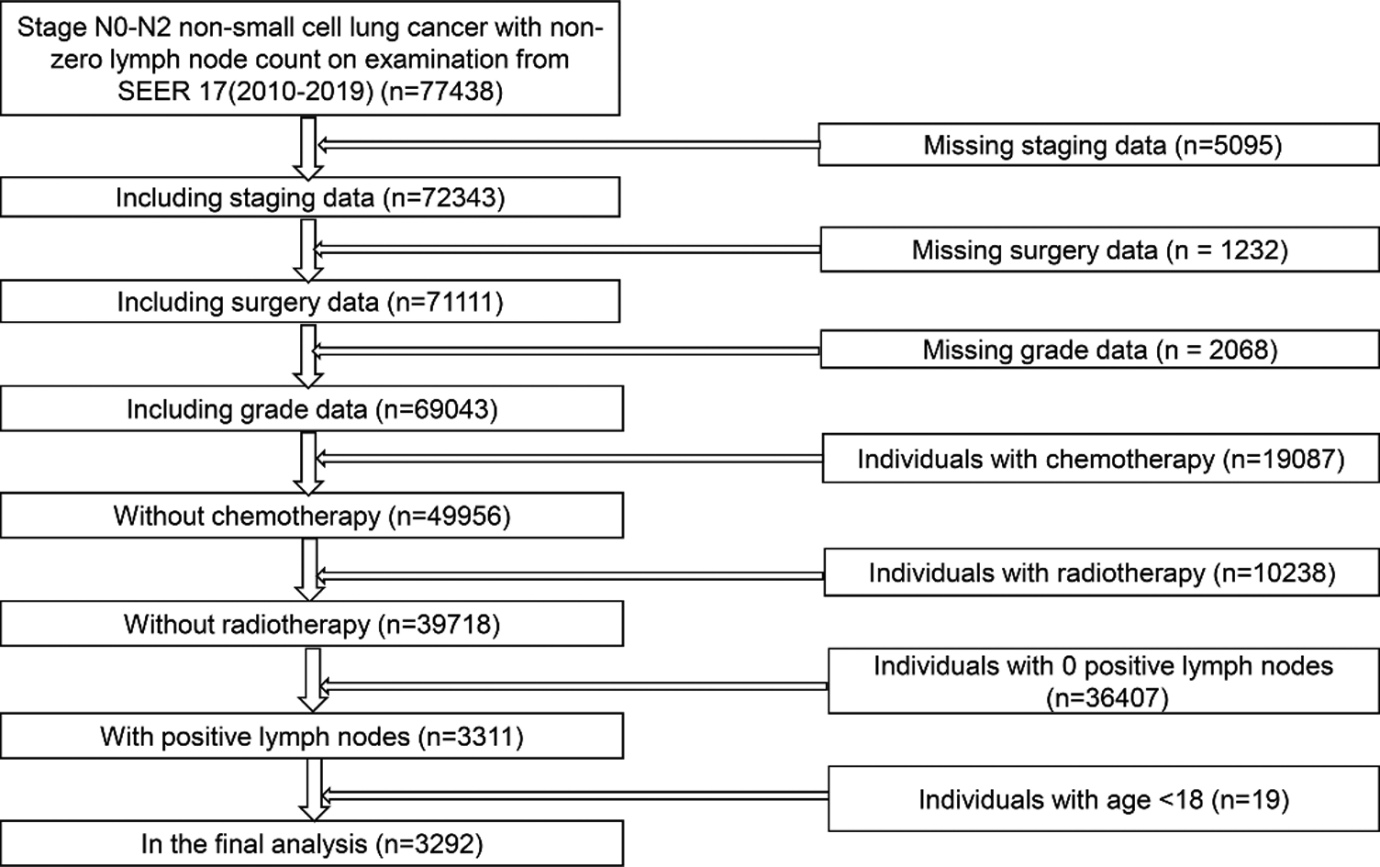
Participants’ screening flowchart.

We meticulously curated data from the SEER 17 Registered Studies database, renowned for its contemporaneousness, encompassing records spanning from 2010 to 2019. Our cohort comprised adult patients diagnosed with NSCLC who underwent surgical excision of the primary tumor, with intraoperative removal of at least 1 lymph node. Histological subtypes considered encompassed adenocarcinoma (ADC), squamous carcinoma, large cell carcinoma, and other subtypes falling under the rubric of NSCLC. Notably, individuals undergoing chemotherapy and radiotherapy were systematically excluded from our analysis (Fig. 1). Exclusions included those receiving chemotherapy or radiotherapy. This exclusion was implemented to minimize the effect of confounding variables on survival outcomes and to ensure that the observed association between LNR and CSS reflects the independent prognostic value of LNR. Including patients with adjuvant treatments could introduce variability in survival outcomes, as chemotherapy and radiotherapy may significantly influence prognosis and obscure the true relationship between LNR and survival. Subsequent to meticulous data curation, patient profiles were meticulously scrutinized and staged in accordance with the rigorous standards delineated by the American Joint Committee on Cancer, as articulated in its 8th edition classification schema. It is imperative to underscore that this study obtained requisite approval from the Ethics Committee of Jiangxi Provincial People’s Hospital, thereby affirming adherence to ethical precepts and regulatory stipulations throughout the investigative process.

### 2.2. Cohort selection

**Figure 2.**
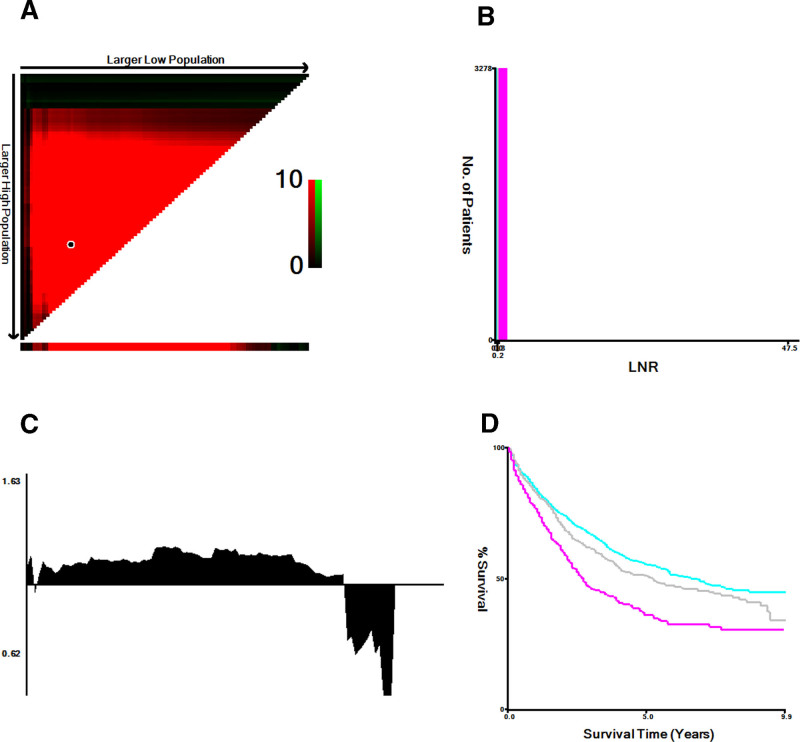
X-tile analysis of cancer-specific survival in patients with stage N1 and N2 non-small cell lung cancer. The optimal cut point occurs at the brightest pixel (green or red). (Critical points in (A) are shown on a histogram of the entire cohort (B), and relative risk (RR) is shown in (C) and plotted on a Kaplan–Meier plot (D).

From the SEER database, we extracted an array of variables including age, sex, race, primary site, grade, histology, T stage, N stage, operation type, the ratio of positive lymph nodes, time, and CSS. Notably, CSS events were defined as deaths attributed to lung cancer. The primary objective of our investigation centered on the stratification of CSS by LNR, while the secondary focus was on delineating LNR-stratified CSS within N1 and N2 stage NSCLC cases. Employing the sophisticated X-tile software, we ascertained the optimal cutoff value for LNR. X-tile is a well-established bioinformatics tool that allows for outcome-based optimization of cutoff values using a statistical approach. Unlike arbitrary or percentile-based methods, X-tile evaluates all possible cutoff points to identify the most statistically significant thresholds by minimizing within-group heterogeneity and maximizing between-group differences. This method is particularly suitable for our study as it effectively stratifies patients based on CSS outcomes, ensuring that the resulting risk groups are both clinically meaningful and statistically robust. By using X-tile, we mitigate the risk of subjective bias in cutoff selection and enhance the reliability of our LNR stratification. Subsequently, patients were categorized into 3 distinct LNR cohorts: LNR ≤ 0.17, 0.17 < LNR ≤ 0.34, and LNR > 0.34 (Fig. 2).

### 2.3. Statistical analysis

In elucidating descriptive statistics, categorical variables were delineated through absolute numbers and proportions, while Gaussian-distributed continuous variables were represented by mean and standard deviation, and non-normally distributed continuous variables were characterized using median, along with the 25th percentile (Q1) and the 75th percentile (Q3). The calculation of CSS entailed the employment of the Kaplan–Meier method, supplemented by the log-rank test for statistical comparisons. To discern independent risk factors for CSS, multivariate Cox regression analysis was conducted, utilizing LNR classification or N stage as covariates. Furthermore, the Akaike information criterion (AIC) was employed to gauge the goodness of fit between models incorporating LNR categorization and those incorporating pN categories. AIC is a statistical measure used to evaluate the relative quality of models for a given dataset, with lower AIC values indicating a better-fitting model. By penalizing the complexity of the model (i.e., the number of parameters), AIC balances model accuracy and parsimony. In our study, the LNR-based model demonstrated a significantly lower AIC value compared to the pN-based model, indicating that LNR provides a superior prognostic fit and utility for cancer-specific survival in NSCLC patients. All analyses were performed using Empower (R) (www.empowerstats.com, X&Y solutions, Inc., Boston, MA) and R version 3.6.3 (http://www.R-project.org). Empower Stats is a statistical software based on the R language for data analysis. The software has powerful data processing functions, as well as comprehensive analysis functions. *P* < .05 was accepted to indicate statistical significance.

## 3. Results

### 3.1. Baseline population characteristics

The delineation of the study’s breadth is illustrated in Table 1. Over the course of the study, a cohort comprising 3292 patients met the predefined eligibility criteria and was consequently admitted into this investigation. Slightly surpassing half of the cohort (52.76%) comprised male individuals. The preponderance of cases (52.22%) manifested the upper lobe of the lung as the principal primary site for NSCLC, with ADC emerging as the predominant histologic subtype (44.17%). Furthermore, the staging distribution revealed that 88.09% and 11.91% of NSCLC cases were classified as N1 and N2, respectively. The median follow-up duration for CSS among patients was 22 months, with interquartile ranges (Q1, Q3) spanning from 8 to 48 months. A total of 1265 patients succumbed to NSCLC during the stipulated follow-up interval.

**Table 1:** Baseline information on participants.

	Mean (SD) Median (Q1–Q3)/N (%)
ELN	13.57 (9.74) 11.00 (7.00–18.00)
LNR	0.28 (1.03) 0.15 (0.08–0.29)
Age	
<65 yr	937 (28.46%)
≥65 yr	2355 (71.54%)
Sex	
Male	1737 (52.76%)
Female	1555 (47.24%)
Race	
White	2748 (83.48%)
Black	257 (7.81%)
Other	287 (8.72%)
Hispanic/Non-His	
Spanish	217 (6.59%)
Non-Spanish	3075 (93.41%)
Year of diagnosis	
2010–2014	1657 (50.33%)
2015–2019	1635 (49.67%)
Primary site	
Upper lobe	1719 (52.22%)
Middle lobe	176 (5.35%)
Lower lobe	1196 (36.33%)
Main bronchus	54 (1.64%)
Other	147 (4.47%)
Grade	
I	422 (12.82%)
II	1412 (42.89%)
III	1403 (42.62%)
IV	55 (1.67%)
Histology	
SCC	961 (29.19%)
ADC	1454 (44.17%)
ADSC	877 (26.64%)
T stage	
T1	984 (29.89%)
T2	1615 (49.06%)
T3	529 (16.07%)
T4	164 (4.98%)
N stage	
N1	2900 (88.09%)
N2	392 (11.91%)
Operation type	
Lobectomy	2747 (83.44%)
Wedge resection	108 (3.28%)
Segmental resection	52 (1.58%)
Pneumonectomy	385 (11.70%)

ADC = adenocarcinoma, ADSC = adenosquamous carcinoma, ELN = examined lymph nodes, LNR = lymph node ratio, SCC = squamous cell carcinoma.

### 3.2. Comparison of survival prediction effects of LNR and N staging

The median lymph node retrieval count stood at 11 (interquartile range [Q1, Q3]: 7, 18), while the median LNR was recorded at 0.15 (interquartile range [Q1, Q3]: 0.08, 0.29). Utilizing X-tile analysis on CSS data culled from the NSCLC cohort in the SEER registry, optimal LNR thresholds of 0.17 and 0.34 were identified, effectively segmenting the entire cohort into low (LNR1 ≤ 0.17; n = 1864 [56.6%]), medium (0.17 < LNR2 < 0.34; n = 846 [25.7%]), and high (LNR3 > 0.34; n = 582 [17.7%]) strata.

**Figure 3.**
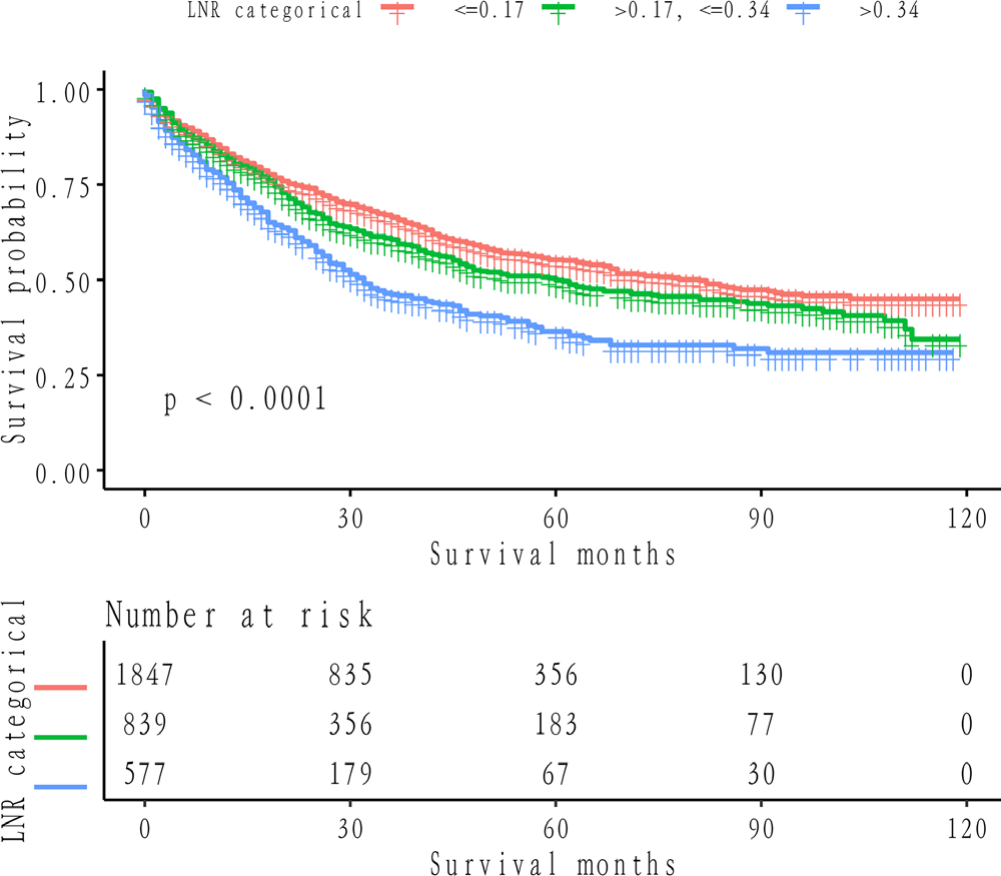
Cancer-specific survival of non-small cell lung cancer patients stratified by LNR. LNR = lymph node ratio.

**Figure 4.**
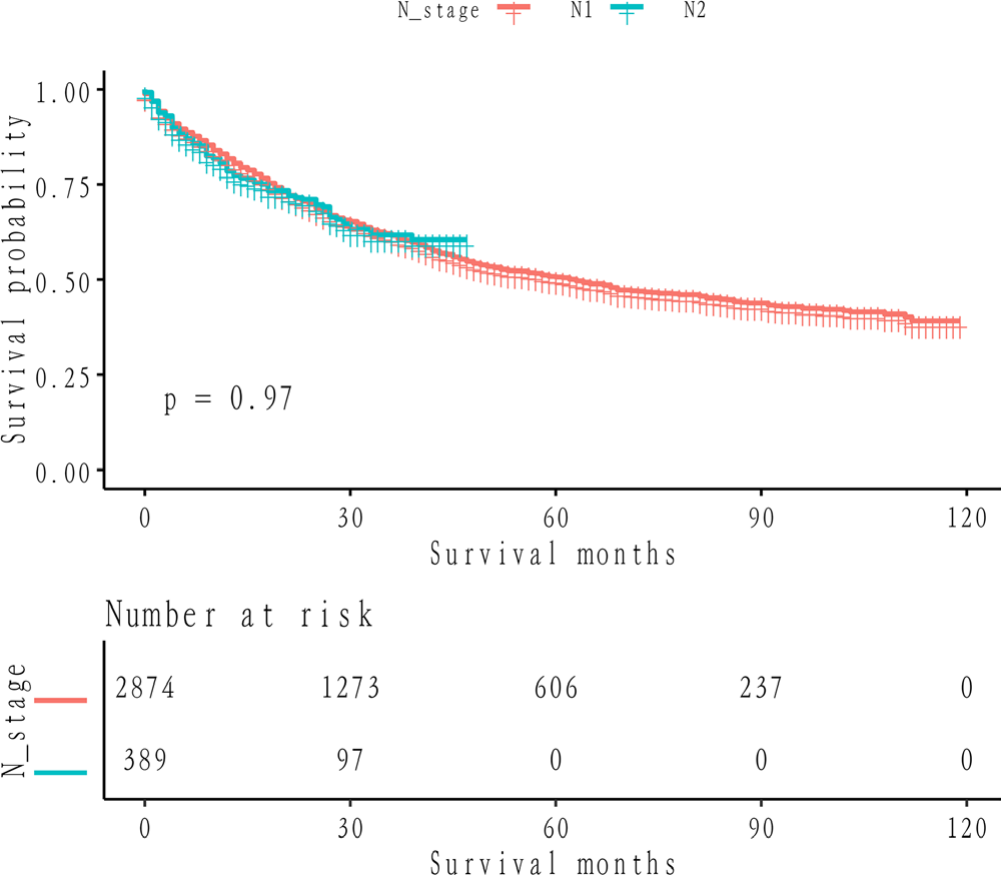
Cancer-specific survival of non-small cell lung cancer patients stratified by N stage.

Kaplan–Meier survival analysis demonstrated a significant improvement in CSS among patients in the low LNR group compared to those in the intermediate or high LNR groups (*P* < .0001). This is visually summarized in Figure 3, which clearly shows the Kaplan–Meier survival curves for the 3 LNR categories (≤0.17, 0.17–0.34, and >0.34). Patients with higher LNR (>0.34) exhibit significantly poorer survival probabilities, supporting the prognostic value of LNR in stratifying patient outcomes (Fig. 3). Importantly, the identification of LNR cutoffs at 0.17 and 0.34 allows for clinically relevant stratification into low-risk (≤0.17), medium-risk (0.17–0.34), and high-risk (>0.34) groups. This stratification has significant clinical implications: Low-risk patients (LNR ≤ 0.17) may require standard postoperative surveillance with routine follow-up. Medium-risk patients (0.17 < LNR ≤ 0.34) represent an intermediate group who may benefit from adjuvant therapies, such as chemotherapy, to reduce recurrence risk. High-risk patients (LNR > 0.34) are at substantially increased risk of poor outcomes and may warrant more aggressive treatment strategies, including intensive adjuvant therapies and closer follow-up. By incorporating LNR into clinical practice, these findings provide a practical tool for improving risk stratification, guiding individualized treatment decisions, and potentially enhancing long-term survival outcomes for NSCLC patients. Notably, the Kaplan–Meier curves illustrating LNR demonstrated superior discriminative potential relative to those based on N stage stratification (LNR *P* < .0001 vs N stage *P* = .91) (Fig. 4). Multivariate Cox regression analysis, with LNR as the focal variable, underscored higher LNR as an independent and substantive harbinger of compromised CSS in the fully adjusted model. The lung cancer-specific mortality rate within the high LNR cohort surpassed that of the low LNR cohort by a factor of 1.72 (95% confidence interval: 1.48, 1.99) with a *P*-value < .0001, while the medium LNR cohort exhibited a 1.20-fold (95% confidence interval: 1.05, 1.37) elevation in mortality compared to the low LNR cohort (*P* = .0060) (Table 2).

**Table 2:** Multivariate regression analysis.

Exposure	Non-adjusted	Adjust I	Adjust II
LNR categorical			
≤0.17	1	1	1
>0.17, ≤0.34	1.17 (1.03, 1.33) 0.0180	1.20 (1.05, 1.37) 0.0065	1.20 (1.05, 1.37) 0.0060
>0.34	1.66 (1.44, 1.91) <0.0001	1.74 (1.51, 2.01) <0.0001	1.72 (1.48, 1.99) <0.0001
Age			
<65 yr	1	1	1
≥65 yr	1.53 (1.34, 1.75) <0.0001	1.52 (1.33, 1.73) <0.0001	1.36 (1.19, 1.55) <0.0001
Sex			
Male	1	1	1
Female	0.75 (0.67, 0.83) <0.0001	0.75 (0.67, 0.84) <0.0001	0.80 (0.71, 0.90) 0.0001
Race			
White	1	1	1
Black	0.93 (0.75, 1.15) 0.5113	1.00 (0.81, 1.23) 0.9930	0.97 (0.78, 1.20) 0.7478
Other	0.94 (0.77, 1.15) 0.5548	0.92 (0.75, 1.12) 0.4072	0.94 (0.76, 1.15) 0.5377
Primary site			
Upper lobe	1	1	1
Middle lobe	0.66 (0.49, 0.88) 0.0044	0.68 (0.51, 0.91) 0.0087	0.77 (0.58, 1.03) 0.0748
Lower lobe	1.01 (0.89, 1.13) 0.9095	1.01 (0.89, 1.14) 0.8941	1.06 (0.94, 1.19) 0.3553
Main bronchus	1.14 (0.75, 1.73) 0.5283	1.28 (0.84, 1.94) 0.2486	0.99 (0.64, 1.53) 0.9625
Other	1.27 (0.99, 1.63) 0.0565	1.32 (1.03, 1.69) 0.0285	1.33 (1.02, 1.71) 0.0320
Grade			
I	1	1	1
II	2.81 (2.18, 3.63) <0.0001	2.61 (2.02, 3.37) <0.0001	2.35 (1.80, 3.06) <0.0001
III	4.13 (3.21, 5.31) <0.0001	3.76 (2.91, 4.84) <0.0001	3.34 (2.57, 4.35) <0.0001
IV	4.45 (2.92, 6.78) <0.0001	4.06 (2.66, 6.20) <0.0001	3.86 (2.52, 5.90) <0.0001
Histology			
SCC	1	1	1
ADC	0.91 (0.80, 1.04) 0.1638	0.96 (0.85, 1.10) 0.5747	1.13 (0.99, 1.29) 0.0818
ADSC	0.64 (0.55, 0.75) <0.0001	0.72 (0.61, 0.84) <0.0001	0.97 (0.83, 1.14) 0.7058
T stage			
T1	1	1	1
T2	1.37 (1.19, 1.57) <0.0001	1.34 (1.17, 1.54) <0.0001	1.20 (1.04, 1.38) 0.0122
T3	2.13 (1.80, 2.51) <0.0001	2.06 (1.74, 2.44) <0.0001	1.78 (1.50, 2.11) <0.0001
T4	2.38 (1.86, 3.05) <0.0001	2.29 (1.78, 2.93) <0.0001	1.81 (1.39, 2.36) <0.0001
N stage			
N1	1	1	1
N2	1.00 (0.82, 1.23) 0.9660	1.05 (0.86, 1.28) 0.6443	1.10 (0.90, 1.35) 0.3404
Operation type			
Lobectomy	1	1	1
Wedge resection	1.07 (0.77, 1.49) 0.6826	1.09 (0.78, 1.52) 0.6005	1.09 (0.78, 1.52) 0.6238
Segmental resection	0.79 (0.46, 1.37) 0.4108	0.74 (0.43, 1.28) 0.2812	0.78 (0.45, 1.35) 0.3676
Pneumonectomy	1.63 (1.39, 1.90) <0.0001	1.68 (1.43, 1.96) <0.0001	1.32 (1.11, 1.57) 0.0019

Non-adjusted model adjust for: none. Adjust I model adjust for: age, sex, race. Adjust II model adjust for: age, sex, race, primary site, grade, histology, T stage, N stage; operation type.

ADC = adenocarcinoma, ADSC = adenosquamous carcinoma, LNR = lymph node ratio, SCC = squamous cell carcinoma.

Comparison of 2 multivariate Cox models incorporating LNR and pN as covariates revealed a lower AIC for the LNR model (LNR vs N stage: 3955.4668 vs 4335.6585), affirming the superior prognostic utility of LNR vis-à-vis N stage for CSS.

## 4. Discussion

In this investigation, we leveraged population-based data to appraise the prognostic significance of the LNR in individuals afflicted with NSCLC. Our findings underscore the autonomy of LNR as a prognostic determinant in both N1 and N2 stage NSCLC cases. Moreover, the delineation of optimal LNR cutoff values via the meticulous X-tile method emerges as pivotal, as it enables the stratification of lymph node-positive NSCLC cohorts into distinct subgroups. This stratification holds profound implications for guiding the discerning selection of clinical treatment modalities. Previous studies on NSCLC have demonstrated the prognostic significance of LNR; however, the reported cutoff values vary due to differences in sample size, methodologies, and patient populations. For instance, Feng et al^[20]^ analyzed data from the SEER database and identified LNR thresholds of 0.21 and 0.38 in stage II NSCLC patients, with higher LNR values associated with significantly worse overall survival. Similarly, our study identified optimal LNR cutoffs of 0.17 and 0.34, which align with the general trends observed in prior research while providing refined, statistically validated thresholds. These findings highlight the clinical utility of LNR for risk stratification and emphasize its potential role in guiding postoperative treatment strategies.

Precision in staging lung cancer is paramount, exerting profound influence on treatment selection, imparting prognostic insights to patients and their kin, and fostering effective discourse among healthcare practitioners, particularly in the context of tailored stratification within clinical trials. While the TNM staging system stands as a cornerstone in oncological practice, ongoing refinements are indispensable to facilitate bespoke patient care. Notably, the intricacies of N staging are multifaceted, influenced by variables such as age, tumor localization, T-staging, extent of lymph node dissection, and the meticulousness of pathological assessment. Moreover, mounting evidence underscores the inherent heterogeneity in prognostic outcomes among NSCLC patients, particularly within the N2 subgroup, even when T, N, and M staging are ostensibly equivalent.^[21–25]^ Some investigations have illuminated the potential prognostic advantages associated with skip N2 metastasis in NSCLC, with studies by Asamura et al^[26]^ advocating for the inclusion of anatomical lymph node localization, metastatic station enumeration, and skip metastasis assessment in prognostic evaluations. However, the prognostic ramifications of skip N2 lymph node metastasis in NSCLC remain contentious; while Tamura et al^[27]^ found no correlation between skip N2 metastasis and survival in N2 NSCLC, others have observed conflicting results. Despite initial indications of prognostic promise in univariate analyses, subsequent multivariate assessments have often failed to validate skip N2 metastasis as an independent prognosticator.^[28]^ The LNR, calculated as the ratio of pathologically positive lymph nodes to the total number of examined lymph nodes, emerges as a salient prognostic metric owing to its incorporation of both the quantity of pathologically positive lymph nodes and the extent of lymph node examination. Widely recognized for its prognostic utility across diverse malignancies, including breast cancer^[29–31]^ and melanoma,^[32]^ LNR assumes similar prognostic significance in the domain of NSCLC. Our investigation corroborates the notion that LNR furnishes supplementary, and potentially more precise, prognostic insights than prevailing nodal staging paradigms.

Lymph node metastases and their distribution among patients afflicted with NSCLC emerge as pivotal determinants influencing both prognosis and therapeutic avenues. While the lymph node status in numerous malignancies typically hinges upon both the anatomical site and the extent of lymph node involvement, the prevailing classification of lymph node status within the TNM system for NSCLC, predominantly factors in the anatomical localization of involvement, overlooking the numerical aspect. Mounting evidence underscores a discernible correlation between the quantity of retrieved lymph nodes (fewer than 10 vs 10 or more) and the prognosis of survival, a dynamic that may precipitate shifts in staging criteria, potentially compromising the precision of survival prognostication.^[31]^ In light of these observations, certain scholars have proposed a refined classification system that stratifies N1 disease into discrete subcategories contingent upon the number of positive lymph node stations.^[33]^ An inherent challenge in gauging the nexus between survival outcomes and the number of positive lymph node stations lies in its intrinsic confounding with the extent of lymph node dissection. Furthermore, the concept of examined lymph node (ELN) count has emerged as an autonomous prognostic determinant in NSCLC patients^[34]^; however, the absence of standardized protocols governing lymph node dissection management and examination underscores an area of ambiguity. The variability in ELN counts across institutions further compounds these challenges, with discrepancies arising from the inclusion of both intact lymph nodes and lymph node fragments in the counts. This variability underscores the dynamic nature of ELN counts, contingent upon the pathological milieu and institutional practices. In light of these potential limitations, the concept of LNR as a prognostic metric in NSCLC has garnered increasing attention, offering a promising avenue for refining prognostication amidst the complexities of lymph node involvement.

Adjuvant chemotherapy stands as a standard recommendation for patients grappling with NSCLC showcasing indications of lymph node metastasis, heralding a notable upsurge in survival rates. LNR emerges as an autonomous prognostic determinant, offering a nuanced approach to staging and bespoke postoperative adjuvant therapies tailored for individuals contending with NSCLC. Those harboring elevated LNR levels confront heightened risks of recurrence, necessitating consideration for aggressive postoperative interventions to fortify their long-term prognosis. Presently, the National Comprehensive Cancer Network guidelines advocate surgical intervention as the principal recourse for individuals clinically staged as I–IIa (T1–2, N0, M0) in NSCLC.^[35]^ Postsurgical modalities entail adjuvant chemotherapy alone for those designated with pathologic stage N0, while the addition of mediastinal radiotherapy supplements adjuvant chemotherapy for those designated with pathologic stage N1 or N2. Nonetheless, the employment of adjuvant chemotherapy is not devoid of peril, with attendant risks of toxicity and adverse reactions. Advanced age, compromised functional status, or the presence of multiple comorbidities may impede patients’ tolerance to adjuvant chemotherapy.^[36]^ Hence, there is a cogent argument for clinicians to contemplate discontinuation of adjuvant therapy in cases where the LNR falls below 0.15.^[37]^ While the advent of validated biomarkers holds promise for the future, the utility of LNR in prognostication remains paramount, affording clinicians a more discerning approach to anticipate recurrences. Such insights empower patients and their kin to tailor their follow-up regimens and chart enduring treatment strategies with greater precision. The identification of LNR thresholds provides a practical tool for risk stratification and prognostic evaluation in NSCLC patients, with clear implications for clinical decision-making. For example: Low LNR group (≤0.17): patients in this group demonstrate favorable survival outcomes and may be suitable for standard postoperative follow-up without the immediate need for adjuvant therapy, thereby minimizing overtreatment. Intermediate LNR group (0.17–0.34): these patients exhibit moderate risk and could benefit from tailored treatment approaches, such as further assessment of additional prognostic factors (e.g., tumor molecular markers or performance status) to determine the need for adjuvant chemotherapy. High LNR group (>0.34): patients in this group are at significantly increased risk of poor survival outcomes. For these individuals, aggressive treatment strategies, including adjuvant chemotherapy and/or radiotherapy, should be strongly considered to improve prognosis. Close postoperative surveillance with shorter follow-up intervals may also be warranted for timely detection of recurrence. These practical applications underscore the value of integrating LNR into current clinical workflows as a complementary tool alongside traditional TNM staging. By enabling more precise risk stratification, LNR facilitates individualized treatment strategies, optimizing therapeutic decisions and improving patient outcomes.

Our investigation bears certain constraints. Our inquiry was circumscribed by the absence of certain covariates within the SEER database, such as comorbidities, performance status assessments, total tumor burden, systemic therapeutic interventions, or subsequent localized treatments. Second, the exclusion of patients who underwent chemotherapy or radiotherapy, although necessary to minimize confounding factors, may have introduced selection bias and limited the generalizability of the findings to all NSCLC patients. Patients receiving adjuvant treatments may exhibit different survival outcomes, and their exclusion may underestimate or overestimate the prognostic value of LNR. Third, the variability in lymph node dissection practices across different institutions could impact the calculation of the LNR. Institutions with more extensive lymph node dissections may identify a greater number of negative lymph nodes, potentially lowering the LNR, whereas limited dissections could lead to overestimation of LNR values. This variability highlights the importance of standardizing lymph node dissection practices to ensure the consistent application of LNR in clinical settings.

Consequently, the veracity of LNR in prognosticating outcomes among patients afflicted with NSCLC mandates further elucidation through prospective investigations. Future research should focus on the following directions to further validate and expand the clinical applicability of LNR in NSCLC management: 1. Standardizing lymph node dissection procedures: Variability in lymph node dissection practices across institutions may influence LNR calculation. Standardized protocols for lymph node sampling and dissection will ensure consistency and improve the comparability of results across studies. 2. Exploring LNR in different NSCLC subgroups: Future studies should investigate the prognostic impact of LNR in specific NSCLC subgroups, such as ADC, squamous cell carcinoma, and early-stage patients. This will help determine whether LNR thresholds need to be adjusted for different histological types or disease stages. 3. Integrating LNR into clinical decision algorithms: Prospective, multicenter trials are needed to evaluate the utility of LNR in guiding postoperative treatment decisions, such as the need for adjuvant chemotherapy or radiotherapy in high-risk patients.

## 5. Conclusions

This study demonstrates that the LNR is a superior prognostic indicator for N1 and N2 stage NSCLC compared to traditional N staging. By integrating metastatic burden and lymph node retrieval extent, LNR addresses key gaps in the TNM system and provides enhanced risk stratification. Clinicians can use LNR to identify high-risk patients who may benefit from intensified adjuvant therapies or tailored follow-up protocols. Further prospective studies are warranted to establish standardized LNR thresholds and validate its integration into clinical practice.

## Acknowledgments

We hereby thank the participants for their time and energy in the data collection phase of SEER project.

## Author contributions

**Conceptualization:** Tao Hu.

**Data curation:** Ying Huang.

**Formal analysis:** Ying Huang.

**Investigation:** Xiaowei He, Ying Huang.

**Methodology:** Xiaowei He, Ying Huang.

**Project administration:** Xiaowei He, Ying Huang.

**Resources:** Xiaowei He.

**Software:** Xiaowei He.

**Supervision:** Tao Hu.

**Validation:** Xiaowei He, Tao Hu.

**Visualization:** Xiaowei He, Tao Hu.

**Writing – original draft:** Xiaowei He, Tao Hu.

**Writing – review & editing:** Xiaowei He, Tao Hu.
